# Microbiota dynamics and source tracing during the growing, aging, and decomposing processes of *Eucommia ulmoides* leaves

**DOI:** 10.3389/fmicb.2024.1470450

**Published:** 2024-12-03

**Authors:** Qiuyu Shao, Chunbo Dong, Yanfeng Han, Yanwei Zhang

**Affiliations:** ^1^Key Laboratory of Development and Utilization of Biological Resources in Colleges and Universities of Guizhou Province/Key Laboratory of Ecology and Management on Forest Fire in Higher Education Institutions of Guizhou Province, Guizhou Education University, Guiyang, China; ^2^Institute of Fungus Resources, College of Life Sciences, Guizhou University, Guiyang, China

**Keywords:** phenological stage, leaf microbiota, root microbiota, taxa overlap, source-sink relationship

## Abstract

*Eucommia ulmoides*, an important tree, faces serious threat to its growth from environmental stress, particularly climate change. Using plant microbes to enhance host adaptation to respond climate change challenges has been recognized as a viable and sustainable strategy. However, it is still unclear how the perennial tree microbiota varies across phenological stages and the links between respective changes in aboveground and belowground niches. Here, we sequenced 27 root and 27 leaf samples of *E. ulmoides* using 16S rRNA and ITS amplicon sequencing techniques. These samples were obtained from the three main phenological stages of leaves, including leaf growing, aging and decomposing stages. Results showed that the diversity, composition, and function of the leaf microbiota of *E. ulmoides* showed more obvious changes at three phenological time points compared to roots. Regarding alpha diversity, the root microbiota showed no difference across three sampling stages, while the leaf microbiota varied with sampling stages. Regarding beta diversity, the root microbiota clustered from different sampling stages, while the leaf microbiota exhibited distinct separation. Regarding composition and function, the dominant taxa and main functions of the root microbiota were the same in three sampling stages, while the leaf microbiota in the decomposing stage was obviously different from the remaining two stages. Additionally, taxa overlap and source-sink relationship existed between *E. ulmoides* microbiota. Specifically, the degree of overlap among root microbiota was higher than that of leaf microbiota in three sampling stages. The bidirectional source-sink relationship that existed between the root and leaf niches varied with sampling stage. During the leaf growing and aging stages, the proportion of microbial members migrating from roots to leaves was higher than the proportion of members migrating from leaves to roots. During the leaf decomposing stage, the migration characteristics of the fungal community between the root and leaf niches maintained the same as in the remaining two stages, but the proportion of bacterial members migrating from leaves to roots was significantly higher than that of members migrating from roots to leaves. Our findings provide crucial foundational information for utilizing *E. ulmoides* microbiota to benefit their host under climate change challenges.

## 1 Introduction

*Eucommia ulmoides*, a deciduous perennial tree belonging to the Eucommiaceae family and *Eucommia* genus, possesses important medicinal, industrial, and ecological value (Yang et al., 2019). The examination of *E. ulmoides* fossils through evolutionary history studies has revealed its emergence approximately 65 million years ago in Zhejiang Province, China (Xu et al., 2021). Subsequently, around 55 million years ago, *E. ulmoides* spread to different regions, including North America, Europe and other Asian countries, and formed more than 10 species, such as *E. montana*, *E. browni*, *E. japonica*, and *E. europaea* (Call and Dilcher, 1997; Xu et al., 2021). However, as a result of environmental changes, these closely related species have been successively extirpated, and only *E. ulmoides* survived as single family, genus, and species of plant (Xing et al., 2019). The Earth currently undergoing rapid climate change, characterized by both warming and localized cooling (Hatfield and Prueger, 2015). Without the implementation of mitigation measures, it is projected that global average surface temperatures will increase by 2.6–4.8°C by the year 2100 (Trivedi et al., 2022). Despite the reduced length of winter in certain regions due to global warming, it is noteworthy that approximately two-thirds of the Earth’s land surface experience subzero temperatures at least once annually (Kazemi-Shahandashti and Maali-Amiri, 2018; Acuña-Rodríguez et al., 2020). Moreover, it is anticipated that the frost frequency and severity will persistently escalate during the transition period from winter to spring in the foreseeable future (Kazemi-Shahandashti and Maali-Amiri, 2018). These environmental stresses present a significant peril to the development, reproductive capacity, and productivity of *E. ulmoides*.

Fortunately, despite the inherent vulnerability of plants to environmental change due to their fixed biological trait, their ability to shape associated microbial communities could provide an extra line of defense against environmental stress (Sawicki et al., 2015; Kashyap et al., 2023). For example, *Burkholderia phytofirmans* has been found to enhance plant cold hardiness by increasing the host’s photosynthetic rate and promoting the production of proline and phenolics under low temperature stress (Ait Barka et al., 2006). Similar to other plants, different niches of *E. ulmoides*, such as the rhizosphere, bark and seed, contain abundant microbes that have been shown to play a crucial role in the host’s growth, health, productivity and synthesis of medicinal compounds (Yang et al., 2019; Dong et al., 2022; Zhang et al., 2023). However, the majority of existing research has only examined the microbiota during a single sampling period, and our understanding of how the microbiota of *E. ulmoides* varies across different phenological phases is currently limited.

Present studies are insufficient to reveal the highly complex and dynamically changing microbiota of perennial tree *E. ulmoides*. In addition, the issue is further complicated by the observation that temporal variations in the plant microbiota differ across niches (Wagner, 2021). Usually, compared to underground organs, plant aboveground organs perceive changes in seasonal or climatic conditions more rapidly and initiate local responses to recruit different microbiota (Trivedi et al., 2022). On this basis, it has been suggested that long-distance communication exists between the aboveground and underground organs of plants, and that climatic stress-induced changes in leaves could also have a cascading effect on root microbiota composition (Hou et al., 2021). Currently, research mostly focuses on local responses of aboveground organ microbiota, while the indirect responses of underground organ microbiota remain inadequately comprehended. Here, we revealed the microbiota dynamics and linkages in the aboveground (leaf) and underground (root) niches of *E. ulmoides* at three time points. These time points were set according to the leaf phenology characteristics of *E. ulmoides* and corresponded to important leaf phenological events, including leaf growing (characterized by fully spread leaves with a yellow-green surface), aging (marked by yellowing and the initiation of leaf abscission) and decomposing (the decomposition and decay of shed leaves returned to the soil environment) stages. We focused on two main questions: (i) How did the microbiota of *E. ulmoides* leaf and root niches differ at the three designated sampling time points? (ii) What were the dissimilarities and correlations observed between the microbiota of *E. ulmoides* leaf and root niches at the three designated sampling time points? This investigation will enhance comprehension regarding the dynamics and stability of the *E. ulmoides* microbiota, which provides important basic information for managing microbes to benefit the host under climate change scenarios.

## 2 Materials and methods

### 2.1 Sample collection and processing

Samples for this study were collected from the Bozhou production region (altitude: 1002.25 m, longitude: E 106°5324′, latitude: N 27°6122′) situated in Guizhou Province, China. In this region, three sample plots of 20 m × 20 m were set up. From each plot, three healthy *E. ulmoides* (approximately 15 years old trees) were chosen, and samples of their leaves and roots were collected in March 2022 (spring), November 2022 (autumn) and January 2023 (winter) during leaf growing, aging and decomposing stages, respectively. The procedure for sampling spring and autumn leaf samples was identical. Specifically, a complete branch was gathered from each plant, situated approximately 10 m above the ground, directly connected to the central trunk. All leaves (10–15 g) from the sampled branch were collected to represent the leaf niche. Decomposing and decaying leaves (10–15 g) of *E. ulmoides* were collected as winter leaf samples from litter collection nets that had been previously laid on the ground for each plant. The laying of litter collection nets was carried out after leaf sample collection was completed in the autumn (leaf aging stage), which ensured that the leaves collected during the decomposing stage were mainly derived from naturally aging and shedding leaves. Collected leaf samples were placed into sterile bags and immediately lyophilized using liquid nitrogen, then stored at −80°C until leaf microbial DNA extraction.

Root sample collection procedures remained consistent throughout the three sampling time points. Specifically, three orientations about 1 m from the *E. ulmoides* trunk were selected, and after removing weeds and topsoil, a mixed sample of soil and roots in the 10–30 cm depth interval was obtained at each orientation using a shovel. The sampling mass for a single orientation was approximately 600 g. Soil and root samples from three orientations were mixed to a total of approximately 1.8 kg. After picking out the *E. ulmoides* fine roots (approximately 1 mm in diameter) from the 1.8 kg soil and root mixture sample and gently shaking to remove loose soil bound to the surface of the fine roots, a sterile soft brush was used to brush the soil from the surface of the fine roots onto a sterile aluminum foil to obtain rhizosphere soil sample. The mass of each rhizosphere soil sample was about 50 g, which were laid out in a well-ventilated and pollution-free room to air dry for physical and chemical properties determination. Root samples after removal of rhizosphere soil represented root niche and they were lyophilized using liquid nitrogen immediately after being individually packed in sterile bags and stored at −80°C until root microbial DNA extraction.

### 2.2 DNA extraction, amplicon sequencing and bioinformatic analysis

After homogenization of leaf and root samples using a sterilized mortar and pestle, microbial DNA was extracted from 0.5 g of homogenized powder using the Magnetic Plant DNA Kit (Tiangen Biotech (Beijing) Co., Ltd.). The bacterial 16S rRNA gene V3-V4 region was amplified by using primers 335F: 5′-CADACT CCTACGGGAGGC-3′ and 769R: 5′-ATCCTGTTTGMTMCC CVCC-3′. The fungal ITS1 region was amplified using primers ITS1F: 5′-CTTGGTCATTTAGAGGAAGTAA-3′ and ITS2: 5′-GCTG CGTT CTTCATCGATGC-3′. All amplification reactions were conducted in a reaction volume of 10 μL, and the specific amplification system, temperature cycling parameters, and high throughput amplification sequencing steps of PCR products were consistent with those described by Shao et al. (2023). The raw reads obtained from sequencing were subjected to quality filtering with Trimmomatic 0.33. Following this, the primer sequences were identified and removed using Cutadapt 1.9.1 software, with a maximum mismatch rate of 20% and a minimum coverage of 80%. The reads from each sample were subsequently merged using Usearch 10 software, with a minimum overlap length of 10 bp, a minimum similarity of 90% allowed in the overlap region, and a maximum of 5 bp mismatched bases. Finally, UCHIME 8.1 was utilized to remove chimeras and acquire reads of high quality. These reads were then denoised using the DADA2 method in QIIME2, resulting in the generation of ASVs. A conservative threshold of 0.005% was applied for ASV filtration. The sequence data has been uploaded to the NCBI Sequence Read Archive database under the BioProject PRJNA952834. Bacterial ASVs were annotated using SILVA (release 138) as the reference database, while fungal ASVs were annotated using and UNITE (release 8.0), both with a confidence threshold of 70%. Abundance tables were created for taxonomic levels including phylum, class, order, family, genus, and species, using species taxonomic information corresponding to each ASV. The functional profiles of bacteria and fungi were inferred using FAPROTAX and FUNGuild databases, respectively (Louca et al., 2016; Nguyen et al., 2016). For fungal function annotation, only taxa with confidence rankings of “Highly probable” and “Probable” were retained.

### 2.3 Acquisition and determination of environmental factors

Environmental factors in this study included sampling region climate data and rhizosphere soil physicochemical data. Climate factor data were acquired from the China Meteorological Data Service Center.^1^ We obtained the average temperature, average rainfall, average relative humidity, and total sunshine duration for January, March, and November from the platform from 2012 to 2021 (Supplementary Table S1), and performed correlation analyses with the microbial data using the average values of the climatic factors for each sampling month in the decade. Soil physicochemical determination methods were mainly referred to “Physical and Chemical Analysis of Soil Properties” (Ren et al., 2019). Specifically, soil pH, available nitrogen (AN) and available phosphorus (AP) were determined using potentiometric, alkali N-proliferation and molybdenum-blue colorimetry methods, respectively, and results were shown in Supplementary Table S2.

### 2.4 Data analyses

Alpha diversity box plots were generated using the ggplot2 package in R 4.1.2, and the differences between sample groups were assessed using the nonparametric Kruskal-Wallis test. Nonmetric multidimensional scaling (NMDS) analysis, permutational multivariate analysis of variance (PERMANOVA), hierarchical clustering analysis and Mantel test analysis were conducted using the vegan package. Specifically, we assessed the beta diversity of both bacterial and fungal communities by calculating Bray-Curtis distance matrices and then ordinated using NMDS. The relative contribution of the niche and sampling stage of *E. ulmoides* on community dissimilarity was tested with PERMANOVA using the Adonis function. Performing hierarchical cluster analysis for microbial community composition and functional data in *E. ulmoides*. Mantel test was used to analyze (based on Spearman’s correlation) the effects of environmental factors on the diversity, composition, function and co-occurrence network topology feature of microbial communities in *E. ulmoides*. When using Mantel test analyses to determine the correlation between community composition and environmental factors, we decided to compute compositional distance matrices at the phylum taxonomy level to improve the stability of our results. When using the Mantel test to determine the correlation between topological features and environmental factors, we used five topological feature parameters, including node number, average degree, edge number, average clustering coefficient, and modularity, to calculate the distance matrix for each sampling point. Specifically, we first performed microbial co-occurrence network analysis based on Spearman’s correlation coefficients (Spearman’s *r* > 0.6 or *r* < −0.6, *p* < 0.05) using the psych and WGCNA packages. The bacterial and fungal ASVs present in 60% of the samples were selected for network analysis. The network visualization was conducted using Gephi, and topological features of the network were also computed. Then, we normalized the data of each topological feature parameter to the range of 0 to 1 by the Min-max normalization method. Finally, we use Euclidean distances to measure the differences between different topological feature parameters and form a distance matrix of these differences for Mantel test analysis.

In addition, intersection plots were generated using the UpSetR and VennDiagram packages. The source-sink relationship of the *E. ulmoides* microbiota across various sampling stages and niches was analyzed using the fast expectation–maximization microbial source tracking (FEAST) method, available at https://github.com/cozygene/FEAST (Shenhav et al., 2019). Specifically, FEAST analyses iteratively estimate the contribution of different source samples to the microbial community of a target sample through an expectation step and a maximization step. In the FEAST framework, each microbial taxon has a “source” and “sink” environment that corresponds to the origin and destination of its migration. However, due to the simultaneous movement of multiple taxa, a multidirectional source-sink relationship between the target and source samples may eventually be presented. This multi-directionality does not mean that individual taxa migrate bidirectionally between different environments, but rather that each environment could serve as a source for some taxa and a sink for other taxa.

## 3 Results

### 3.1 Diversity dynamics of *Eucommia ulmoides* microbiota

The 16S rRNA amplification sequencing of 54 samples (3 plots × 3 plants × 3 stages × 2 niches) resulted in the generation of 8,643,799 raw reads, with 7,959,308 high quality reads (ranging from 106,422 to 156,791 reads per sample), and the formation of 30,646 ASVs (with an ASV range of 275–2,438). Similarly, the ITS amplification sequencing of the same 54 samples yielded 8,641,036 raw reads, with 8,428,600 high quality reads (ranging from 150,593 to 158,335 reads per sample), and the formation of 7,556 ASVs (with an ASV range of 215–722). Rarefaction curves were constructed for each individual sample, and they generally approached saturation, suggesting that the sequencing depth adequately captured the majority of microbial community diversity (Supplementary Figure S1).

**Figure 1:**
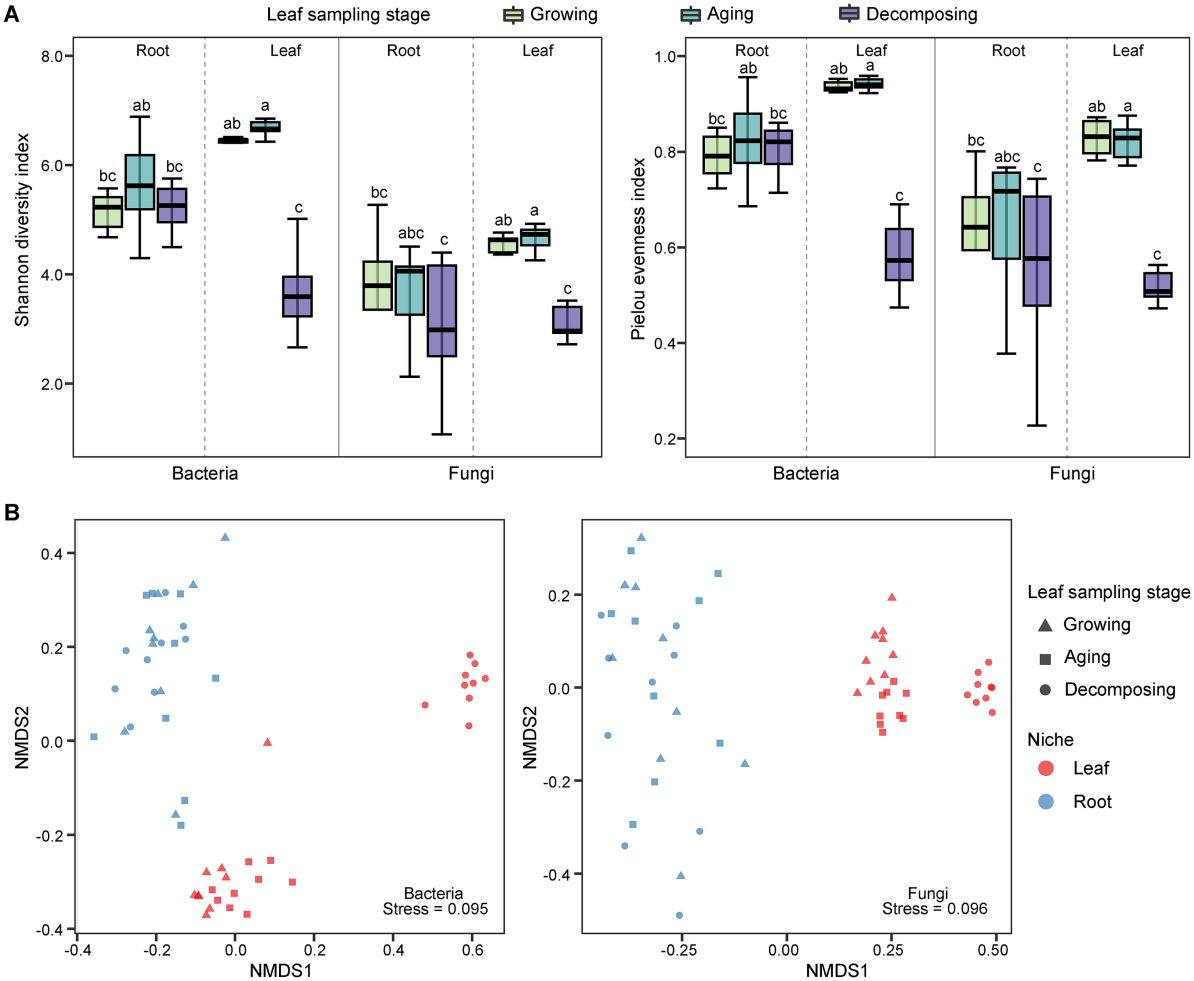
Microbial community diversity of *E. ulmoides* at different sampling stages and niches. **(A)** Alpha diversity boxplot of *E. ulmoides* microbial community. Diversity data were analyzed by the nonparametric Kruskal-Wallis test, and significant differences between groups were indicated by lowercase letters. **(B)** NMDS ordination at the ASV level based on Bray-Curtis distance matrix (*n* = 54).

The alpha diversity of *E. ulmoides* microbiota was assessed using the Shannon diversity index and pielou evenness index (Figure 1A). Compared to root microbes, the diversity of leaf-associated microorganisms exhibited greater variability at different sampling stages in *E. ulmoides*. There were no significant differences in bacterial or fungal community diversity among the root samples across the three sampling stages. However, the leaf samples exhibited distinct variations depending on the sampling stage. The overall microbial diversity in the leaves was highest during the leaf aging stage, followed by the leaf growing stage, and lowest at the leaf decomposing stage and differed significantly from the remaining two stages. Beta diversity exhibited similar patterns to alpha diversity. Specifically, the microbial communities of root samples were clustered at the ASV level during three stages, while the microbial communities of leaf samples showed a distinct separation (Figure 1B). The PERMANOVA results further revealed that both the sampling niche and sampling stage contributed to the dynamics of the *E. ulmoides* microbiota, which determined 9.86 and 9.42% of bacteria community variance and 15.95 and 7.78% of fungi community variance, respectively (Table 1). These findings suggest that the sampling niche has a stronger influence on the *E. ulmoides* microbiota than the sampling stage. In addition, the interaction between the sampling niche and sampling stage explained community variation significantly higher than the single effects, explaining 28.02 and 30.87% of the variation in bacteria and fungi communities, respectively (Table 1).

**Table 1:** Influence of host niche, sampling stage and their interaction on *E. ulmoides* microbiota based on PERMANOVA.

Variables	Bacteria				Fungi			
	Df^a^	F^b^	*R*^2^^c^ (%)	Pr (>F)^d^	Df^a^	F^b^	*R*^2^^c^ (%)	Pr (>F)^d^
Niche	1	5.69	9.86	0.001	1	9.87	15.95	0.001
Stage	2	2.65	9.42	0.001	2	2.15	7.78	0.001
Niche × stage	5	3.74	28.02	0.001	5	4.29	30.87	0.001

a Degree of freedom.

b The test value of the model.

c Degree of explanation for differences between samples by grouping mode.

d Significance *p* value, when the value was less than 0.05 indicates high confidence in the test.

### 3.2 Composition and functional dynamics of *Eucommia ulmoides* microbiota

**Figure 2:**
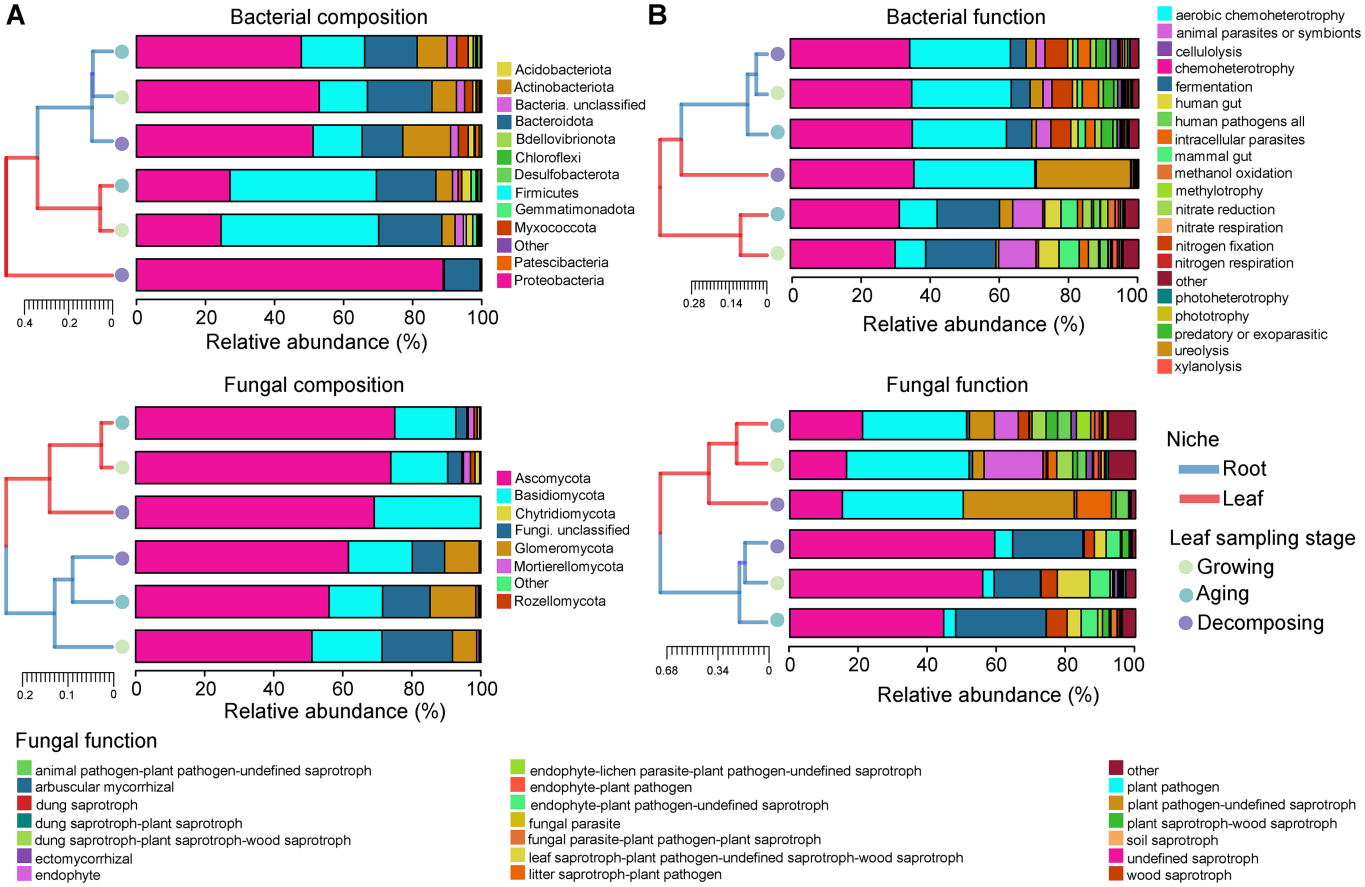
Microbial community composition and function of *E. ulmoides* at different sampling stages and niches. **(A)** Histogram of hierarchical clustering of *E. ulmoides* microbial community composition (phylum level). Low abundance phyla with less than 1% of total sequences in all samples were classified as “Other.” **(B)** Histogram of hierarchical clustering of *E. ulmoides* microbial community function. Histograms showed only the abundance top 20 functional taxa in all samples, and the remaining functional groups were classified as “Other.” Clustering between sample groups was performed based on the Bray-curtis weighting method. Close distance and short branch lengths between the two sample groups indicate high similarity in their microbial community composition or function.

Analysis of microbial community composition (Figure 2A) and functional (Figure 2B) hierarchy clustering demonstrated clear and separate clustering between root and leaf niches. Generally, the clustering branches between *E. ulmoides* root niches were short for three sampling stages, indicating a high degree of similarity in their microbial community composition and function. During the leaf growing, aging and decomposing stages, root bacterial communities were primarily composed of Proteobacteria, accounting for 52.68, 47.48, and 50.85% of the total abundances, respectively. The main functional groups (relative abundance >10%) observed were chemoheterotrophy (34.81, 34.97, and 34.27%) and aerobic chemoheterotrophy (28.58, 27.10, and 28.91%). Similarly, root fungal communities during the leaf growing, aging and decomposing stages were predominantly composed of Ascomycota, representing 51.01, 56.03, and 61.63% of the total abundances, respectively. The main functional groups included undefined saprotroph (55.80, 44.49, and 59.29%) and arbuscular mycorrhizal (13.38, 26.14, and 20.32%).

Clustering branches observed between leaf niche samples were longer compared to root samples, indicating that the composition and function of the leaf microbial community associated with *E. ulmoides* is more dynamic than that of the root microbial community (Figures 2A,B). During the leaf growing and aging stages, leaf bacterial communities were primarily composed of Firmicutes, accounting for 45.22 and 41.78% of the total abundances, respectively. The main functional groups included chemoheterotrophy (30.04 and 31.27%), fermentation (20.14 and 17.88%), animal parasites or symbionts (10.75 and 8.60%) and aerobic chemoheterotrophy (8.80 and 10.89%). Similarly, leaf fungal communities during the leaf growing and aging stages were predominantly composed of Ascomycota, representing 73.89 and 75.02% of the total abundances, respectively. The main functional groups included plant pathogen (35.44 and 30.04%), endophyte (17.00 and 6.93%) and undefined saprotroph (16.41 and 21.09%). During the leaf decomposing stage, either the bacterial or fungal composition and function of the leaves were clustered in a single branch, suggesting the *E. ulmoides* leaf microbial community at this time very different from the other two stages. Specifically, the leaf bacterial and fungal communities at decomposing stage were dominated by Proteobacteria (88.82%) and Ascomycota (69.11%), respectively. The primary bacterial functions included chemoheterotrophy (35.45%), aerobic chemoheterotrophy (34.73%) and ureolysis (27.05%), and main fungal functions involved plant pathogen (34.99%), plant pathogen-undefined saprotroph (32.06%), undefined saprotroph (15.17%) and litter saprotroph-plant pathogen (10.02%).

### 3.3 Co-occurrence network pattern dynamics of *Eucommia ulmoides* microbiota

**Figure 3:**
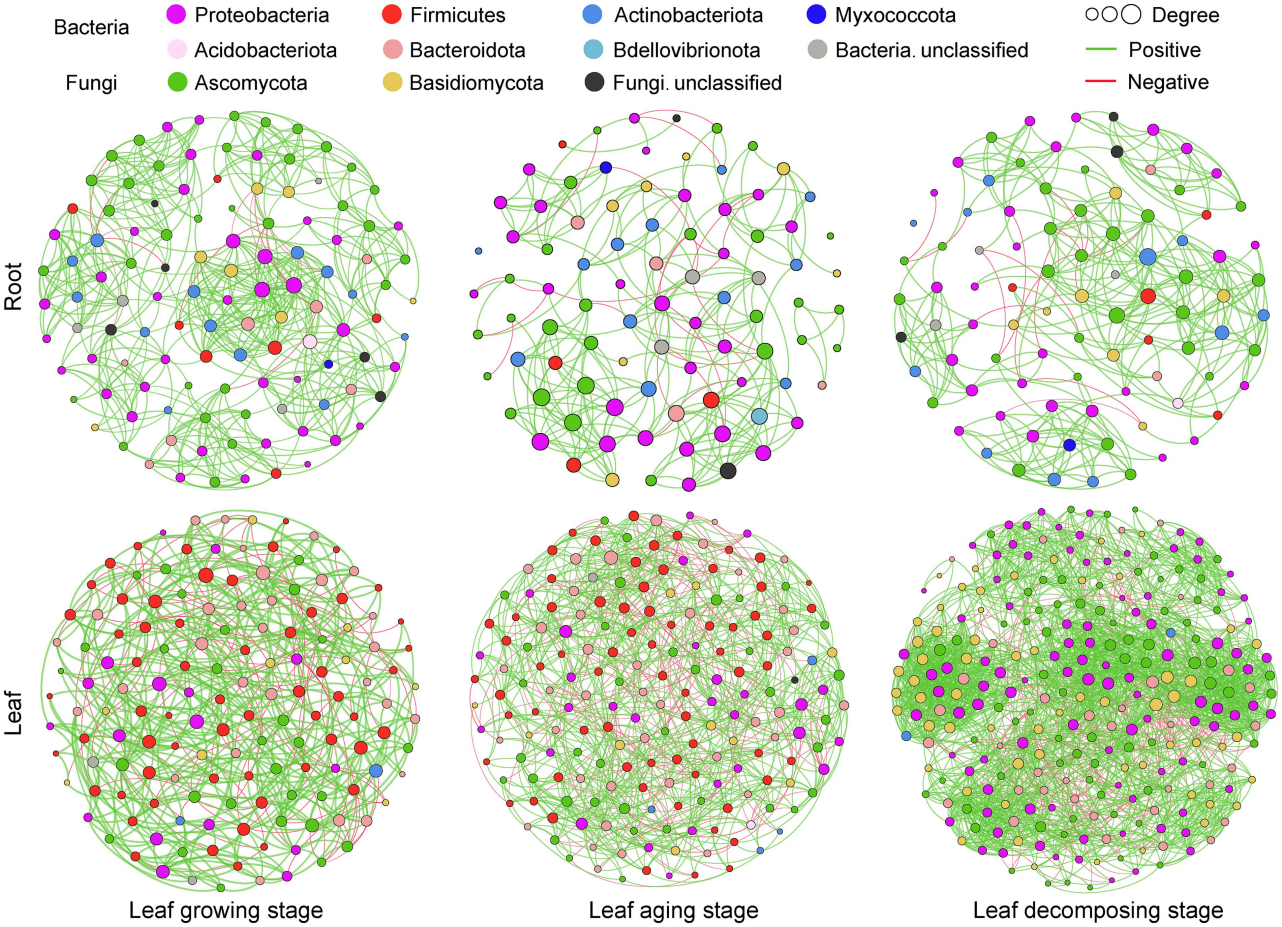
Co-occurrence network of bacterial and fungal ASVs in different sampling stages and niches of *E. ulmoides*.

Same thresholds were employed to construct microbial co-occurrence networks for various sampling stages and niches of *E. ulmoide*, facilitating a comparison of similarities and differences among them (Figure 3; Table 2). Bacterial nodes predominantly dominated each microbial co-occurrence network associated with *E. ulmoide*. Moreover, bacterial taxa exhibited a higher average degree and marginally lower average clustering coefficient compared to fungi, suggesting a greater complexity of bacterial taxa and a stronger clustering tendency of fungal taxa within the *E. ulmoide* microbial community. Noticeably, modularity index (Newman, 2006) values of the *E. ulmoide* microbial co-occurrence network varied between 0.76 and 0.96 across different sampling stages and niches, indicating a high degree of modularity in all co-occurrence networks. Furthermore, irrespective of the sampling stage and niche, the *E. ulmoide* microbial co-occurrence network exhibited a higher proportion of positive correlation compared to negative correlation.

**Table 2:** Topological features of interkingdom co-occurrence networks of *E. ulmoides* bacterial and fungal communities at different sampling stages and niches.

Niche	Sampling stage	Nodes (B/F^a^)	Average degree^b^ (B/F^a^)	Edges (positive/negative)	Average clustering coefficient^c^ (B/F^a^)	Modularity^d^
Root	Leaf growing stage	114 (70/44)	9.81 (10.10/9.34)	559 (542/17)	0.65 (0.65/0.65)	0.78
	Leaf aging stage	88 (53/35)	6.25 (6.92/5.23)	275 (251/24)	0.51 (0.55/0.45)	0.81
	Leaf decomposing stage	90 (51/39)	7.13 (6.49/7.97)	321 (304/17)	0.62 (0.61/0.63)	0.76
Leaf	Leaf growing stage	145 (108/37)	8.30 (8.51/7.70)	602 (483/119)	0.35 (0.35/0.36)	0.81
	Leaf aging stage	189 (141/48)	10.36 (10.54/9.83)	979 (753/226)	0.37 (0.36/0.39)	0.96
	Leaf decomposing stage	269 (135/134)	19.87 (20.59/19.15)	2,673 (2,429/244)	0.55 (0.58/0.52)	0.79

a Bacteria/fungi.

b Refers to the average number of connections of all nodes in the network, and higher values indicate a more complex network.

c Indicates the possibility of interconnection between neighbor nodes of nodes in the network, reflecting the tendency of forming tight groups among nodes.

d Refers to the phenomenon of nodes grouping in the network, where connections between nodes within these groups are tight and connections between groups are relatively sparse, and higher values indicate a tendency for network nodes to differentiate into different groups.

Complexity and clustering degree of the *E. ulmoide* microbial co-occurrence network varied with host niche. The network complexity was characterized by the node number, the average degree and the edge number together, and the network clustering degree was characterized by the average clustering coefficient. Specifically, the microbial co-occurrence network in the leaf niche of *E. ulmoide* exhibited higher complexity and lower aggregation compared to the root niche. The main evidence for this conclusion was that the node number and edge number of leaf network were always higher than those of root network at all sampling stages, but the average clustering coefficient of leaf network was usually lower than that of root network. Additionally, except during the growing stage, the average degree of leaf network was also higher than that of the root network. Complexity and clustering degree of the *E. ulmoide* microbial co-occurrence networks also varied dynamically on the sampling stage. Notably, the complexity and aggregation of microbial networks within the leaf niche demonstrated a progressive increase from leaf growing, aging to decomposing processes. Conversely, the root niche demonstrated the highest microbial network node number, average degree, edge number and average clustering coefficient during the leaf growing stage.

### 3.4 Influence of environmental factors on *Eucommia ulmoides* microbiota

**Figure 4:**
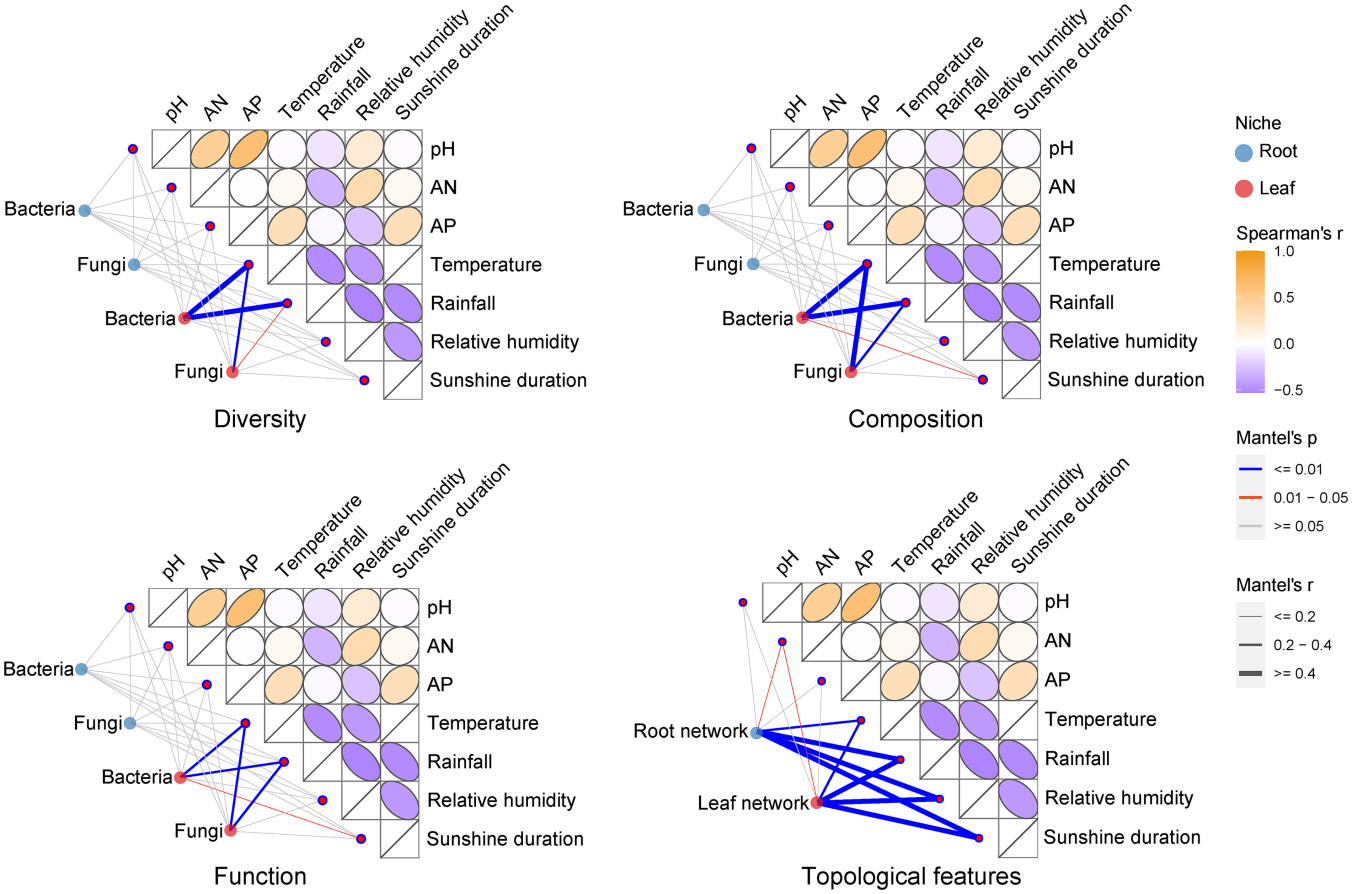
Effects of climatic factors (temperature, rainfall, relative humidity and sunshine duration) and physical and chemical properties of rhizosphere soil (AP, AN, and pH) on *E. ulmoides* microbiota. The climate factor data were monthly average climate data for the research region over the past 10 years, representing average temperature, rainfall, relative humidity, and sunshine duration for three sampling periods. Thus, there were three representative points in time for the climate data. Soil physicochemical data were actual measurements of soil samples gathered from nine sampling points in sequence over the three sampling stages, resulting in a total of 27 data points. This difference in data points was consistent with the experimental design because the long-term averages of the climate data provide a better representation of climatic conditions across sampling stages, while the diversity of the soil data reflects the spatial heterogeneity of soil characteristics within the research region. The correlation between the diversity (including, ACE richness estimator index, Shannon diversity index and Pielou’s evenness index), composition (phylum level), function and co-occurrence network topology feature (including, node number, average degree, edge number, average clustering coefficient and modularity) distance matrices of the *E. ulmoides* microbial community and the distance matrices of the environmental factors were determined using the Mantel test (based on Spearman’s correlation).

We performed Kruskal-Wallis tests on environmental factors from different sampling stages in the sampling region (Supplementary Table S3). The results showed that there were no significant differences (*p* > 0.05) in the rhizosphere soil physicochemical indicators of pH, AN, and AP in different sampling stages. Climate factors including temperature, relative humidity and sunshine duration, except for rainfall, varied at a significant level (*p* < 0.05) across sampling stages. Using Mantel test analysis, we compared the differences in the effects of soil physicochemical and climatic factors on *E. ulmoides* microbiota (Figure 4). Climatic factors had stronger influence on the diversity, composition and function of *E. ulmoides* microbiota, especially leaf microbial communities, than soil physicochemical properties. Specifically, temperature and rainfall were significantly correlated with bacterial diversity and fungal composition, diversity, and function in the leaf niche, and temperature, rainfall and sunshine duration were significantly correlated with bacterial composition and function in the leaf niche, whereas these climatic factors exhibited no significant effect on bacterial and fungal composition, diversity, and function in the root niche. Among the three soil physicochemical factors, only AN had a significant effect on the topological features of the co-occurrence network of root and leaf microbiota in *E. ulmoides* at different sampling stages, while none of these factors had a significant effect on the diversity, composition, and function of the *E. ulmoides* microbiota. These results imply that the microbiota dynamics of *E. ulmoides* in different sampling stages were mainly driven by climatic factors.

### 3.5 Source tracing analysis of *Eucommia ulmoides* microbiota

**Figure 5:**
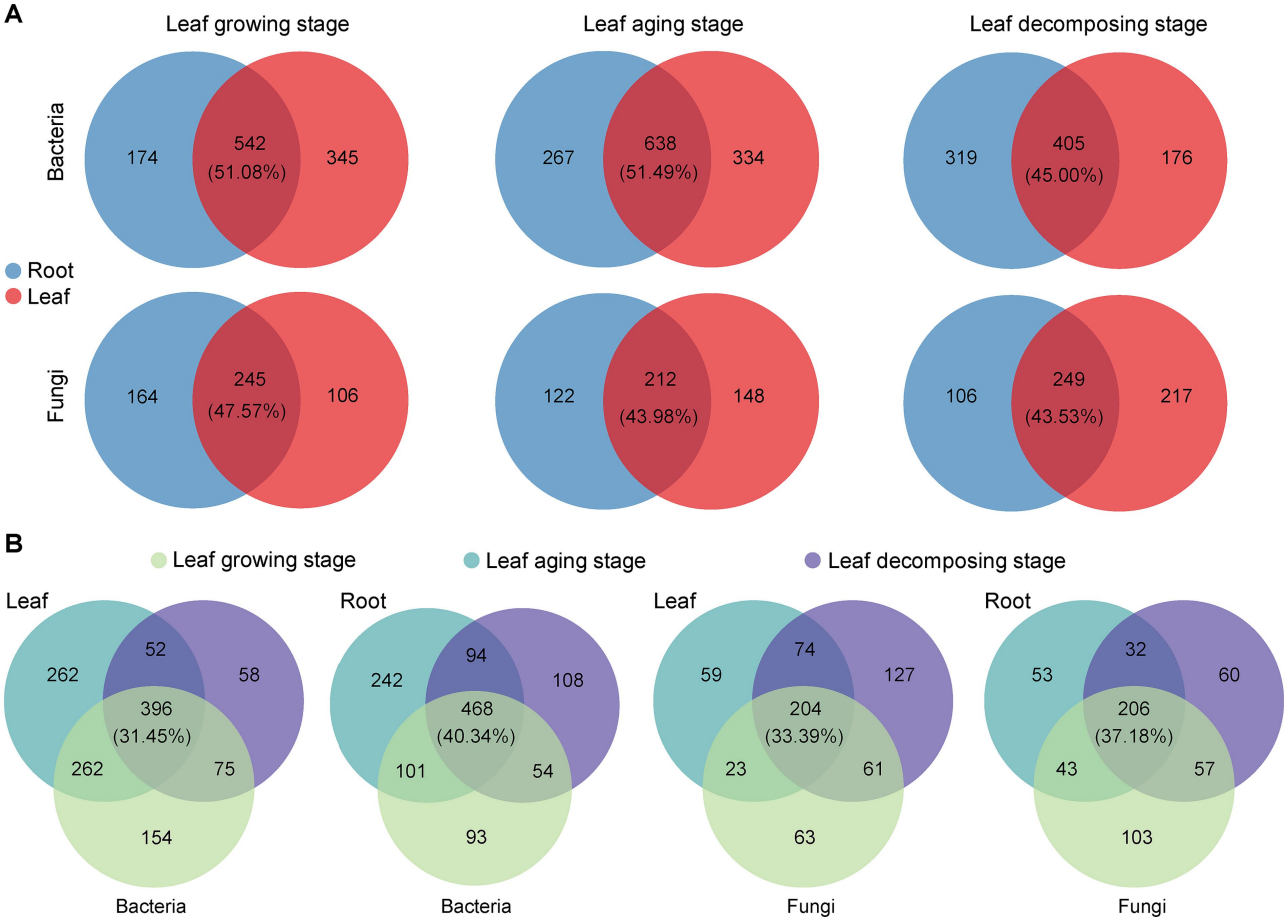
Genus taxonomic level differences in *E. ulmoides* microbial communities across sampling stages and niches. **(A)** Venn analysis of *E. ulmoides* microbial communities at different niches in three sampling stages. **(B)** Venn analysis of *E. ulmoides* microbial communities at different sampling stages for root and leaf niches.

Intersection analysis visually depicted the taxa that were shared and unique among *E. ulmoide* microbial communities in various sampling stages and niches. During leaf growing, aging and decomposing processes, the root and leaf niches of *E. ulmoide* contained a large number of shared microbial genera, exceeding the number of unique taxa (Supplementary Figure S2). Bacterial communities contained 298 shared genera, with the top three genera in relative abundance being *Pseudomonas*, *Massilia*, and *Sphingomonas*, respectively. Fungal communities had 131 shared genera, and the top three in relative abundance, excluding unclassified genera, were *Cladosporium*, *Spirosphaera*, and *Lachnum*, respectively. More specifically, there was obvious overlap between the microbial communities inhabiting root and leaf niches of *E. ulmoide*, regardless of the sampling stage (Figure 5A). Bacterial taxa exhibited a higher degree of overlap between these two niches compared to fungi. During the stages of leaf growing, aging and decomposing in *E. ulmoide*, bacteria existed in 51.08, 51.49, and 45.00% overlap between root and leaf niche in that order, while fungi existed in 47.57, 43.98, and 43.53% overlap, respectively. In addition, the microbial communities of *E. ulmoide* also exhibited significant overlap across various sampling stages within the same niche (Figure 5B). Compared to leaves, a greater degree of microbial overlap was observed among root samples at three sampling stages. Overlap degree of bacteria and fungi was 40.34 and 37.18% in root niche and 31.45 and 33.39% in leaf niche, respectively. These findings suggest that *E. ulmoide* microbes from different niches and different phenological stages within the same niche may be important sources for each other.

**Figure 6:**
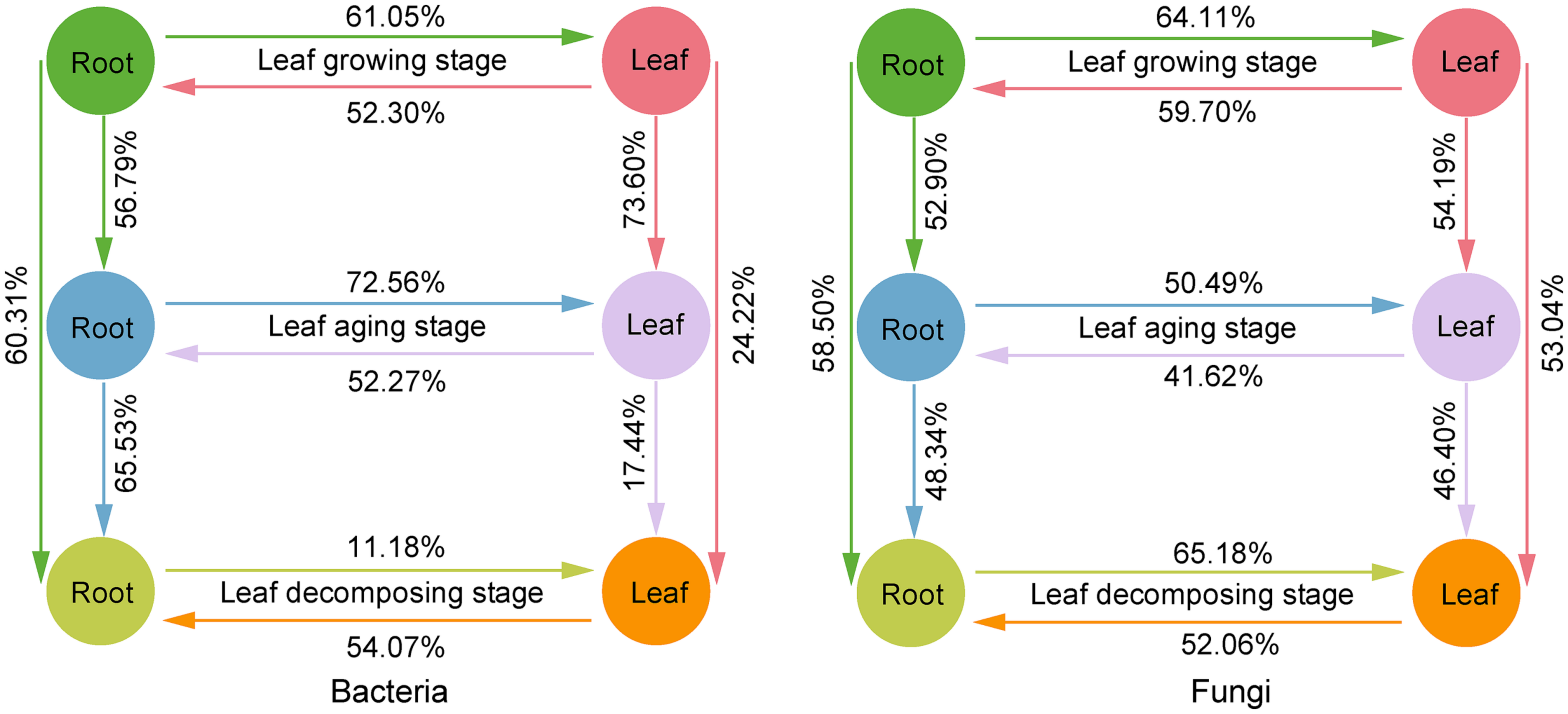
Source-sink relationship between *E. ulmoides* microbiota of different sampling stages (leaf growing, aging and decomposing stages) and niches (root and leaf niches) based on FEAST analysis. In FEAST analysis, the source refers to the environment that provides microbial taxa to other environments and serves as the origin of microbial migration. The sink refers to the environment that receives other environmental microbial taxa and serves as the destination for microbial migration. Here, source or sink environment was represented by different colored circles. The migration of microbes from source to sink environments was indicated by unidirectional arrows. The contribution of the source environment to the microbial community of the sink environment was indicated by percentage values. The existence of a bidirectional source-sink relationship between root and leaf niches resulted from the fact that root and leaf could serve as both source and sink for different microbial taxa, and did not indicate bidirectional migration of a single microbial taxa between root and leaf niches.

Based on FEAST analysis, source-sink relationships were observed between *E. ulmoide* microbiota at different sampling stages in the same niche (Figure 6). The connectivity between microbes in the root niche was generally greater than in the leaf niche during the three sampling stages. Specifically, 60.31 and 58.50% members of the root bacterial and fungal communities in the decomposing stage were from root niche in the growing stage and 65.53 and 48.34% members from root niche in the aging stage, respectively. However, only 24.22 and 53.04% members of the leaf bacterial and fungal communities during the decomposing stage were from leaf niche during the growing stage and 17.44 and 46.40% from leaf niche during the aging stage, respectively. In addition, bidirectional source-sink relationships between the *E. ulmoide* root and leaf niche microbiota were observed, with source-sink values varying depending on the sampling stage (Figure 6). During the growing, aging and decomposing stages, 52.30, 52.27, and 54.07% members of the root bacterial community originated from leaves, and 59.70, 41.62, and 52.06% members of the root fungal community were sourced from leaves, respectively. Conversely, 61.05, 72.56, and 11.18% members of the leaf bacterial community were derived from roots, and 64.11, 50.49, and 65.18% members of the leaf fungal community originated from roots, respectively.

## 4 Discussion

### 4.1 *Eucommia ulmoides* leaf microbiota exhibits greater dynamism than root microbiota across different phenological stages

Plant phenology refers to the phenomenon of regular changes in plant growth in response to seasonal climate variation (Mohamed et al., 2018; Hu et al., 2019; Shigyo et al., 2019). Investigating the dynamic changes in plant microbiota with phenology has positive implications for future microbial management to enhance host adaptation to climate change. The diversity, composition and function of the *E. ulmoides* leaf microbiota exhibited greater variability across three phenological time points compared to the root microbiota. This observation aligns with the widely accepted notion that the seasonal dynamics of aboveground plant microbiota are more pronounced than those occurring belowground (Bahram et al., 2022; Howe et al., 2023). On one hand, the aboveground portion of plant is an open system, and the microbes associated with it originate from multiple sources (Zhu et al., 2022). Particularly, the presence of open stomata and wounds on leaves creates opportunities for colonization by external airborne microbes derived from aerosols, insects or other plants (Xiang et al., 2021; Trivedi et al., 2022; Zhu et al., 2022). On the other hand, arbor leaf traits are usually more sensitive to phenological or seasonal climate change than roots, such as leaf thickness, chemical composition, and area, which are highly variable across leaf developmental stages (Wang and Wang, 2015). Noticeably, among the three phenological time points examined in our study, a strong difference was observed in the *E. ulmoides* leaf microbiota during the leaf decomposing stage compared to the other two stages. This difference could potentially be attributed to the selective colonization of specific microbes facilitated by the unique chemical composition of the leaf at this stage, and the abscission of the leaf back into soil environments (Chen et al., 2020).

In addition, we quantified the relative effects of sampling stage and niche on the *E. ulmoides* microbiota. Our findings revealed that the influence of niche on the *E. ulmoides* microbiota was greater than that of sampling stage. This finding contradicts the results of a previous study that utilized maize, an annual plant, as the subject (Xiong et al., 2021). Specifically, maize developmental stage had a stronger influence on host microbiota than niche, which was attributed to the sampling stage effects representing the interaction of both climatic characteristics (such as temperature, humidity and light) and plant niche characteristics (such as leaf size and chemical composition) (Xiong et al., 2021; Gao et al., 2021). In fact, the variation in microbiota caused by plant niche is also the result of the combination between niche selection filtering and environmental factors with spatial structure (Hu et al., 2019). Vertical stratification of microbial communities has been demonstrated in tree species with tall trunks and dense foliage, where community composition and diversity differ significantly between underground and aboveground niches (Harrison et al., 2016; Bahram et al., 2022). For the canopy alone, changes in microclimatic conditions, leaf morphology and leaf chemistry are enormous and could act as ecological filters leading to variation in microbial communities (Ishii et al., 2008; Harrison et al., 2016). Generally, temperature and light are the primary environmental factors that strongly influence on aboveground niche microbes, while soil physicochemical properties are probably the most important environmental factors determining microbes in the often completely shaded belowground niche (Ishii et al., 2008, Harrison et al., 2016). In our study, it was similarly observed that climatic factors had stronger effects on the microbiota of aboveground niche of *E. ulmoides* compared to the belowground niche. However, the effects of soil physicochemical properties on the diversity, composition and function of belowground niche microbiota were insignificant. This phenomenon might be explained by the fact that the rhizosphere soil physicochemical properties of perennial trees changed insignificantly during different phenological stages, providing a relatively stable habitat environment for the root microbiota and thus weakening the influence on the microbial communities.

### 4.2 Commonalities and identities of the microbial co-occurrence network in *Eucommia ulmoides* across different phenological stages and niches

Considerable progress has recently been made in researching complex microbial communities, in part due to advances in analytical methods. Particularly, co-occurrence network analysis, which distills the complexity of microbial communities into a network, could contribute to reveal community structure (Barberán et al., 2012; Toju et al., 2018; Yeakel et al., 2020). The overall structure of *E. ulmoides* microbial co-occurrence networks exhibited certain resemblances across sampling stages and niches, which were dominated by bacterial nodes and positive correlations, together with high modularity. The similarity observed in microbial co-occurrence networks may be partly attributed to the fact that they all belong to sub-networks of the same plant host. High abundance, wide distribution, and rapid growth potential of bacteria in natural environments may be important reasons for their dominance of *E. ulmoides* microbial networks (Huang et al., 2021). In addition, high modularity may explain another part of the overall structural similarity of the network. Generally, a high degree of modularity could alleviate the impact of microbial taxa disappearance on its belonging modules and other parts of the network (Zhong et al., 2022).

Complexity and aggregation degree within the *E. ulmoides* microbial co-occurrence network varied obviously by niche and sampling stage. Generally, a higher network complexity is associated with a greater aggregation degree (Yuan et al., 2021; Wang et al., 2023). However, the topological characteristics showed that in comparison to the root niche, the microbial co-occurrence network present in *E. ulmoides* leaf niche had higher complexity and lower aggregation. Network aggregation was found to decrease with increasing environmental stress (Hernandez et al., 2021). More specifically, under high-stress environmental conditions, the microbes may be more capable to increase individual survival opportunity by reducing species interdependence through the niche differentiation strategy, which results in communities exhibiting a lower clustering tendency (Li et al., 2023). Accordingly, the low aggregation of leaf microbial communities then may be related to the prolonged exposure of leaves to more severe environmental conditions, such as intense UV radiation, desiccation, and sharp temperature fluctuations, compared to roots. The high complexity of leaf microbial networks may be explained by the following reason. Leaves serve as the primary organs for photosynthesis and material production in plants, synthesizing various nutrients such as sugars, organic acids, and amino acids (Kruger and Volin, 2006). These compounds can be released into the surrounding environment through processes such as leaf leaching or decay, thereby attracting rich microbes. Similar findings have been stated in previous studies, indicating that microbial co-occurrence networks exhibit greater complexity when available nutrient species rich in environment (Meng et al., 2022). The topological characteristics also showed that the complexity and aggregation of *E. ulmoides* leaf microbial network gradually increased from leaf growing to aging and then to decomposing processes. The emergence of this result may be explained by changes in microbial habitat area, and the larger microbial habitats are more likely to accommodate larger numbers of microbes (Meyer et al., 2022). Although the exact leaf area data were not measured in our study, aging leaves always had a larger microbial habitat than growing leaves. In contrast, the root microbial network exhibited the highest level of complexity and aggregation during the leaf growing stage. This observation may be explained by the strongest leaf photosynthetic activity in this period, with the maximum amount of organic matter can be synthesized and transported downwards to roots via the phloem, consequently attracting microbes to inhabit the root niche (Kruger and Volin, 2006).

### 4.3 *Eucommia ulmoides* microbiota in different phenological stages and niches closely linked to each other

Differences in plant microbiota across sampling stages and niches have long been recognized (Wagner, 2021; Fu et al., 2023). However, some studies have also highlighted that plants have stable microbial interaction partners, and these microbe-plant associations can recur across various environments (Junker and Tholl, 2013; Trivedi et al., 2020). In our study, we observed the presence of taxa overlap and bidirectional source-sink relationships within the microbiota of *E. ulmoides* roots and leaves, regardless of the sampling stage. Specifically, bacterial taxa consistently overlapped more than fungal communities between the two niches of *E. ulmoides* root and leaf, regardless of the phenological stage. Fungi have more sensitivity to environmental changes, whereas the rapid growth potential of bacteria helps them to adapt to new environmental conditions, which may contribute to the fact that bacterial taxa have a stronger ability to share plant niches than fungal taxa (Shao et al., 2023). It should be noted in particular that the bidirectional source-sink relationship observed between the microbiota of the root and leaf niches of *E. ulmoides* was caused by the fact that root and leaf could simultaneously serve as source and sink for different microbial taxa, and did not indicate bidirectional movement of specific taxa. During the leaf growing and aging stages, the bidirectional source-sink relationship between the microbial communities in the root and leaf niches of *E. ulmoides* showed that the proportion of members migrating from roots to leaves was higher than the proportion of members migrating from leaves to roots. This result may be related to the following reasons. Plants transport water and nutrients from the roots to the leaves mainly through transpiration. This upward flow of water and nutrients through xylem vessels provides a natural pathway for the migration of root niche microbes to the leaves (Liu et al., 2019; Zhu et al., 2022). In contrast to the unidirectional upward transport mechanism of xylem from root to leaf, plant phloem can transport organic matter synthesized by leaves to multiple parts of the plant such as roots, flowers, and fruits, which diminishes the migration of leaf microbes to the roots. During the leaf decomposing stage, 54.07 and 52.06% members of the root bacterial and fungal communities were from leaves and 11.18 and 65.18% members of the leaf bacterial and fungal communities were from roots, respectively. The steps of microbial migration from leaves to roots may be that old leaves decompose and decay allowing their microbial members to disperse and return to the soil environment, and then into root tissues through newly emerging roots or wounds (Wagner, 2021; Zhu et al., 2022). Microbial migration from roots to leaves may be related to microbes with metabolite substrate preferences (Zhalnina et al., 2018). The degree of preference for the components of *E. ulmoides* decay leaves may determine the size of the proportion of microbial taxa that migrate from roots to leaves. Interestingly, the bacterial community during the decomposing stage exhibited a predominant migration from leaves to roots, while from roots to leaves it showed a disadvantageous migration. The possible reasons for this phenomenon may be as follows. The migration of bacteria from leaves to roots could be enhanced in the soil environment using rainwater as a medium (Xu et al., 2020). In contrast, bacterial migration from underground roots to decaying leaves was extremely challenging. For example, specific bacteria require fungal hyphae as mediators to accomplish migration (Hassani et al., 2018). In addition, taxa overlap and source-sink relationships also existed in *E. ulmoides* microbiota from different sampling stages at the same niche. The degree of overlap and connectivity of root microbiota was generally higher than in leaf microbiota during three stages, which may be due to the more dynamic leaf environment and more diverse sourcing pathways for leaf microbial communities (Bai et al., 2015; Howe et al., 2023).

Currently, a major challenge in plant microbial research is the enormous species composition of microbial communities, as thousands of community members imply millions of possible pairwise interactions (Xue et al., 2023). This complexity is further magnified when we consider transiently present members. Classifying microbes into specific taxa based on their contribution to community stability and their abundance could be a useful approach to reveal the dynamics and migration patterns of arboreal plant microbial communities from complex relationships. Traditionally, microbial community studies have focused mainly on abundant taxa, as they are generally considered the most active participants in biogeochemical cycles, occupying core ecological niches (Jiao et al., 2019; Liang et al., 2020). However, recent studies have increasingly emphasized the ecological importance of rare taxa (Ji et al., 2020; Pascoal et al., 2021). On the one hand, rare taxa can interact more frequently and closely than abundant taxa, which is essential for maintaining microbial community stability (Li et al., 2024). On the other hand, rare taxa tend to be more metabolically active than abundant taxa, enhancing the functional redundancy of the community and increasing resilience to environmental changes or disturbances (Jousset et al., 2017). In our study, we used the overall microbial community data for FEAST analysis to ensure that the results comprehensively represented the linkages between the *E. ulmoides* microbiota across different phenological stages and niches. However, in future studies, distinguishing between abundant and rare taxa may help capture more detailed dynamics and migration patterns of arboreal plant microbial communities.

## Data Availability

The datasets presented in this study can be found in online repositories. The names of the repository/repositories and accession number(s) can be found at: https://www.ncbi.nlm.nih.gov/, BioProject PRJNA952834.

## Footnotes

^1^ http://data.cma.cn
