# Supplementary material for: Microbiota dynamics and source tracing during the growing, aging, and decomposing processes of Eucommia ulmoides leaves
Source: Front Microbiol. 2024 Dec 3;15:1470450. doi: 10.3389/fmicb.2024.1470450 (PMC11649662; doi:10.3389/fmicb.2024.1470450)
Supplement: Supplementary file 1 [file Table_1.docx]

**Supplementary Table S1.** Climate data for each sampling stage from 2012 to 2021 in the *E. ulmoides* sampling region.

| **Climatic factor** | **Sampling month** | **2012** | **2013** | **2014** | **2015** | **2016** | **2017** | **2018** | **2019** | **2020** | **2021** | **Decade average** |
| --- | --- | --- | --- | --- | --- | --- | --- | --- | --- | --- | --- | --- |
| Average temperature (℃) | January | 0.53 | 3.28 | 5.22 | 5.14 | 3.31 | 5.91 | 3.13 | 3.47 | 5.17 | 2.97 | 3.81 |
|  | March | 9.38 | 13.64 | 10.32 | 10.67 | 10.91 | 8.92 | 13.11 | 10.65 | 12.12 | 11.73 | 11.15 |
|  | November | 9.29 | 10.94 | 9.60 | 12.11 | 10.10 | 10.53 | 9.37 | 10.27 | 10.43 | 8.57 | 10.12 |
| Average rainfall (mm) | January | 42.71 | 15.81 | 28.01 | 44.48 | 57.24 | 37.67 | 63.05 | 51.63 | 60.35 | 35.91 | 43.69 |
|  | March | 31.93 | 47.16 | 64.14 | 34.39 | 60.48 | 33.22 | 57.45 | 29.77 | 35.15 | 17.40 | 41.11 |
|  | November | 30.08 | 56.86 | 41.23 | 94.20 | 39.76 | 20.50 | 29.90 | 30.50 | 26.32 | 26.19 | 39.55 |
| Average relative humidity (%) | January | 82.82 | 77.89 | 70.48 | 81.73 | 84.73 | 84.14 | 81.76 | 87.66 | 85.76 | 81.71 | 81.87 |
|  | March | 73.63 | 67.99 | 76.77 | 81.16 | 77.81 | 82.47 | 71.98 | 75.14 | 77.02 | 80.89 | 76.49 |
|  | November | 82.49 | 80.13 | 86.13 | 83.93 | 83.81 | 80.02 | 79.56 | 84.21 | 79.54 | 84.06 | 82.39 |
| Total sunshine duration (h) | January | 91.15 | 102.40 | 109.42 | 84.83 | 90.80 | 93.16 | 87.49 | 82.67 | 87.80 | 94.27 | 92.40 |
|  | March | 141.52 | 156.70 | 136.85 | 138.82 | 143.09 | 117.72 | 147.46 | 136.98 | 138.78 | 131.98 | 138.99 |
|  | November | 88.01 | 93.11 | 79.70 | 95.49 | 97.57 | 98.77 | 92.85 | 90.83 | 105.56 | 85.48 | 92.74 |
