# Supplementary material for: Microbiota dynamics and source tracing during the growing, aging, and decomposing processes of Eucommia ulmoides leaves
Source: Front Microbiol. 2024 Dec 3;15:1470450. doi: 10.3389/fmicb.2024.1470450 (PMC11649662; doi:10.3389/fmicb.2024.1470450)
Supplement: Supplementary file 2 [file Table_2.docx]

**Supplementary Table S2.** Physical and chemical data of rhizosphere soil for each sampling stage. Values represent mean ± standard deviation.

| **Sampling stage** | **Plot** | **Plant** | **Sample code** | **pH** | **AN (mg/kg)** | **AP (mg/kg)** |
| --- | --- | --- | --- | --- | --- | --- |
| Leaf aging stage | Plot 1 | Plant 1 | Repeat 1 | 5.46±0.06 | 166.25±4.37 | 29.95±2.62 |
|  |  | Plant 2 | Repeat 2 | 5.30±0.09 | 194.69±5.05 | 5.76±0.57 |
|  |  | Plant 3 | Repeat 3 | 5.19±0.02 | 155.31±12.37 | 14.85±0.26 |
|  | Plot 2 | Plant 1 | Repeat 4 | 5.33±0.05 | 150.94±11.30 | 32.83±2.60 |
|  |  | Plant 2 | Repeat 5 | 5.39±0.03 | 129.06±7.14 | 77.65±2.05 |
|  |  | Plant 3 | Repeat 6 | 6.15±0.14 | 135.63±25.13 | 45.13±3.84 |
|  | Plot 3 | Plant 1 | Repeat 7 | 4.80±0.07 | 125.78±21.84 | 4.74±0.39 |
|  |  | Plant 2 | Repeat 8 | 4.79±0.05 | 150.94±26.25 | 15.63±1.15 |
|  |  | Plant 3 | Repeat 9 | 4.79±0.02 | 120.31±10.72 | 16.96±1.00 |
| Leaf decomposing stage | Plot 1 | Plant 1 | Repeat 1 | 5.2±0.06 | 157.50±4.38 | 18.41±1.56 |
|  |  | Plant 2 | Repeat 2 | 4.96±0.02 | 127.97±5.51 | 11.03±0.67 |
|  |  | Plant 3 | Repeat 3 | 4.99±0.02 | 171.72±2.19 | 14.82±0.40 |
|  | Plot 2 | Plant 1 | Repeat 4 | 5.29±0.02 | 137.81±12.37 | 43.84±2.04 |
|  |  | Plant 2 | Repeat 5 | 5.54±0.02 | 84.22±10.34 | 64.89±5.24 |
|  |  | Plant 3 | Repeat 6 | 5.95±0.14 | 98.44±5.05 | 36.19±1.45 |
|  | Plot 3 | Plant 1 | Repeat 7 | 4.74±0.05 | 88.59±4.19 | 1.66±0.37 |
|  |  | Plant 2 | Repeat 8 | 4.84±0.02 | 101.72±7.47 | 15.79±0.58 |
|  |  | Plant 3 | Repeat 9 | 4.79±0.02 | 98.44±15.57 | 16.08±1.68 |
| Leaf growing stage | Plot 1 | Plant 1 | Repeat 1 | 5.65±0.11 | 195.78±6.56 | 35.88±2.06 |
|  |  | Plant 2 | Repeat 2 | 5.09±0.12 | 144.38±13.13 | 16.14±0.77 |
|  |  | Plant 3 | Repeat 3 | 5.18±0.03 | 150.94±5.05 | 39.60±4.26 |
|  | Plot 2 | Plant 1 | Repeat 4 | 5.20±0.04 | 130.16±7.47 | 65.73±4.68 |
|  |  | Plant 2 | Repeat 5 | 5.14±0.05 | 126.88±11.01 | 78.80±19.54 |
|  |  | Plant 3 | Repeat 6 | 6.18±0.10 | 137.81±24.75 | 52.35±2.38 |
|  | Plot 3 | Plant 1 | Repeat 7 | 4.68±0.03 | 80.94±5.05 | 17.13±0.42 |
|  |  | Plant 2 | Repeat 8 | 4.74±0.02 | 84.22±2.19 | 15.46±5.48 |
|  |  | Plant 3 | Repeat 9 | 4.83±0.03 | 95.16±2.19 | 21.47±4.76 |
