# Supplementary material for: Microbiota dynamics and source tracing during the growing, aging, and decomposing processes of Eucommia ulmoides leaves
Source: Front Microbiol. 2024 Dec 3;15:1470450. doi: 10.3389/fmicb.2024.1470450 (PMC11649662; doi:10.3389/fmicb.2024.1470450)
Supplement: Supplementary file 3 [file Table_3.docx]

## Supplementary Tables

**Supplementary Table S3.** Kruskal-Wallis test results for environmental factors for each sampling stage. Values represent mean ± standard deviation.

| **Environmental factor** | **Leaf decomposing stage** | **Leaf growing stage** | **Leaf aging stage** | **P value** |
| --- | --- | --- | --- | --- |
| pH | 5.15±0.40 | 5.18±0.47 | 5.24±0.44 | 0.768 |
| AN (mg/kg) | 118.49±31.57 | 127.36±36.57 | 147.66±23.33 | 0.224 |
| AP (mg/kg) | 24.75±19.75 | 38.06±23.29 | 27.06±23.08 | 0.234 |
| Temperature (℃) | 3.81±1.58b | 11.15±1.51a | 10.12±0.99a | 0.000 |
| Rainfall (mm) | 43.69±14.98 | 41.11±15.37 | 39.55±21.78 | 0.431 |
| Relative humidity (%) | 81.87±4.81a | 76.49±4.49b | 82.39±2.39a | 0.006 |
| Sunshine duration (h) | 92.40±8.13b | 138.99±10.10a | 92.74±7.31b | 0.000 |

P value indicates significance, when the value was less than 0.05 meaning that the environmental factors differed significantly between sampling stages. Different mall letters indicate significant differences between stages for that parameter
