# Supplementary figures and images for: Microbiota dynamics and source tracing during the growing, aging, and decomposing processes of Eucommia ulmoides leaves
Source: Front Microbiol. 2024 Dec 3;15:1470450. doi: 10.3389/fmicb.2024.1470450 (PMC11649662; doi:10.3389/fmicb.2024.1470450)

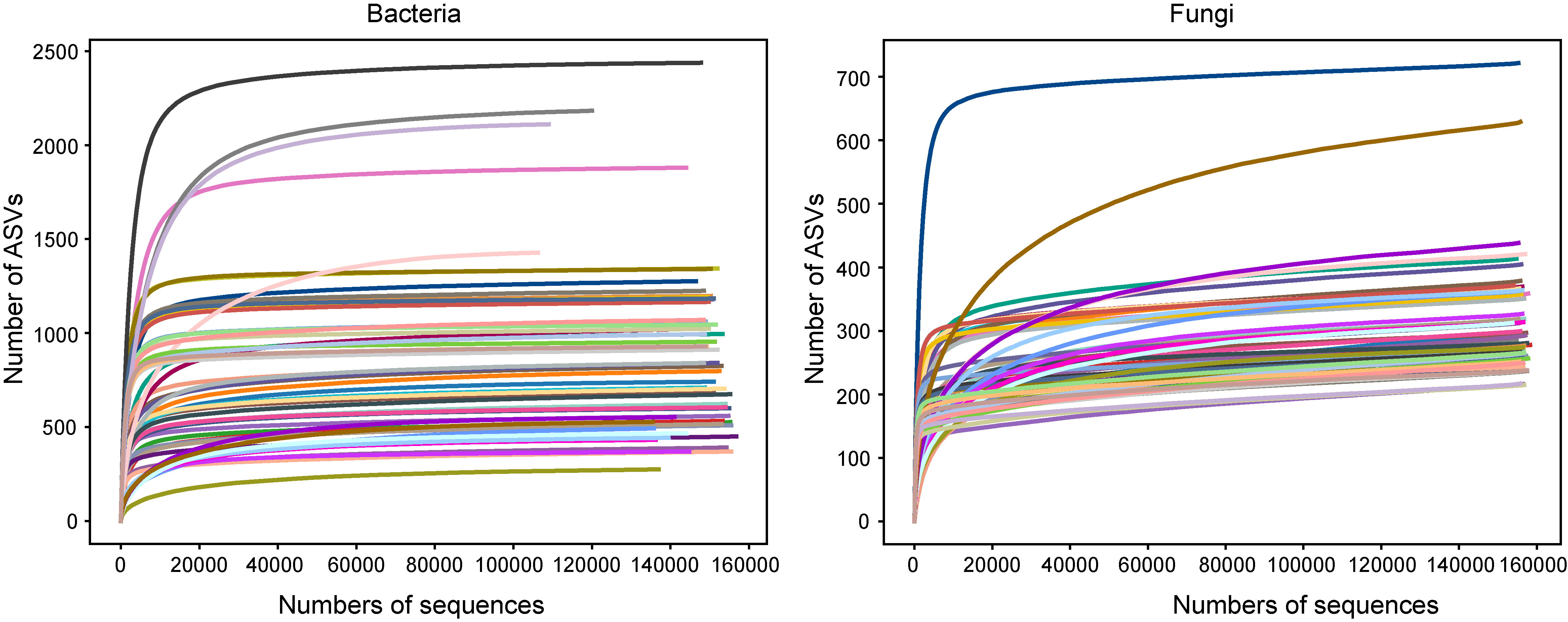

Supplement: Supplementary file 4 [file Image_1.tif]

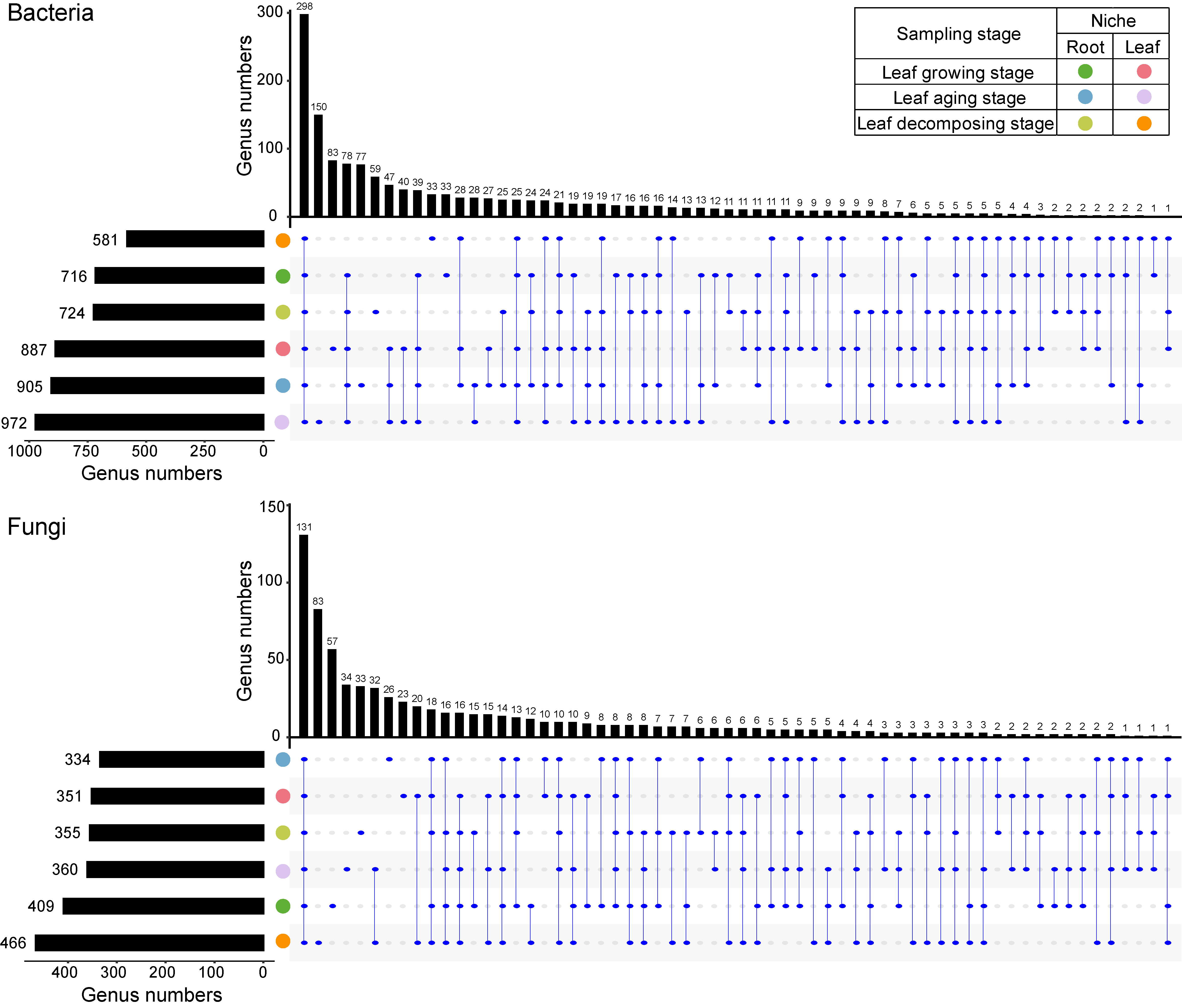

Supplement: Supplementary file 5 [file Image_2.tif]
